# E6-Specific Detection and Typing of Human Papillomaviruses in Oral Cavity Specimens from Iranian Patients

**DOI:** 10.18869/acadpub.ibj.21.6.411

**Published:** 2017-11

**Authors:** Hadi Razavi Nikoo, Abdollah Ardebili, Mehrdad Ravanshad, Fatemeh Rezaei, Ali Teimoori, Sayyad Khanizadeh, Mohammad Hassan Pouriayevali, Mehdi Ajorloo

**Affiliations:** 1 Laboratory Science Research Center, Golestan University of Medical Sciences, Gorgan, Iran; 2 Department of Microbiology, Faculty of Medicine, Golestan University of Medical Sciences, Gorgan, Iran; 3 Department of Virology, Faculty of Medical Sciences, Tarbiat Modares University, Tehran, Iran; 4 Health Research Institute, Infectious and Tropical Diseases Research Center, Ahvaz Jundishapur University of Medical Sciences, Ahvaz, Iran; 5 Hepatitis Research Center, Khorramabad University of Medical Sciences, Lorestan, Iran; 6 Department of Hepatitis and AIDS, Pasteur Institute of Iran, Tehran, Iran

**Keywords:** Real-time PCR, Genotyping, Iran

## Abstract

**Background:**

Detection and quantification of human Papillomavirus (HPV) genome in oral carcinoma play an important role in diagnosis, as well as implications for progression of disease.

**Methods:**

We evaluated tissues from 50 esopharyngeal cancers collected from different regions of Iran for HPV E6 using the two type-specific primers sets. *E6* gene of HPV genotypes was amplified by specific primers. The sensitivity of PCR assay was analyzed and determined using HPV-DNA-containing plasmids. Real-time PCR was utilized to determine the prevalence and HPV viral load in patients with oral cavity squamous cell carcinoma.

**Results:**

Eighteen (36%) specimens were positive for HPV. Among the 18 positive specimens, 10 showed HPV-18 (55.55%), and 8 specimens were positive for HPV-11 (44.44%). Of the 18 infected specimens, 6 (33.32%) and 12 (66.65%) were identified as high-titer and low-titer viral load, respectively.

**Conclusions:**

The PCR-based assay, developed in the current study, could be used for HPV detection, quantification, and genotyping in epidemiological and clinical studies.

## INTRODUCTION

Human papillomavirus (HPV), is small, dsDNA virus and is related to a variety of clinical conditions, ranging from benign to malignant tumors and cancers such as oral cavity squamous cell carcinoma (OCSCC)[1]. Two distinct pathways and risk factor groups have been found to be responsible for OCSCC: the first is environmental/behavioral factors such as smoking and/or alcohol consumption, especially when combining together, and the second risk factor is HPV chronic infection[2]. Current studies have demonstrated that HPV plays a key role in the development of OCSCC unrelated to smoking and/or alcohol consumption[3]. Several other studies have shown and confirmed the etiologic role of HPV in OCSCCs[4,5].

HPV-16, -18, -6, -11, and -33 have been indicated to be the most important and prevalent genotypes associated with the oral cavity carcinoma. HPV-16 and HPV-18 have been confirmed to be associated with head and neck carcinoma[5]. HPV E6 and E7 oncoproteins have essential function in the cervical as well as orophararyngeal carcinogenesis[6]. These proteins deregulate cell cycle by inactivating the critical cellular proteins such as P53 and retinoblastoma, which results in cell immortalization and the increased risk of malignant transformation[6]. Hence, the expression of viral oncogenes in tumor might indicate the capability of HPV to develop OCSCC[7].

Currently, the prognostic value of HPV infection in OCSCC has been understated, and numerous studies have reported conflicting results. However, HPV detection and typing in OCSCC specimens is one of the most important developments in oncology[8]. There is now a great interest in using PCR-based methods in epidemiological studies to detect the prevalence of pathogens, including oncogenic HPV types more efficiently[9,10]. Detection and quantification of the HPV DNA in OCSCC are important for diagnosis and evaluation of the lesion progression[11]. PCR-based methods have been shown to have high sensitivity, specificity, and broad-spectrum detection of HPVs[12,13]. The aim of this study is to evaluate tissues from 50 esophageal cancers, collected from different regions of Iran, for HPV DNA. We designed two type-specific primers sets based on the DNA sequence of *E6* gene that allow the identification of high-risk as well as low-risk genotypes. The sensitivity of this PCR assay was determined and obtained using HPV DNA-containing plasmids. Furthermore, we report a real-time analysis in order to assess the prevalence rate and the viral load of HPV in patients with OCSCC.

## MATERIALS AND METHODS

### Patients

Participants in this study were 50 patients with histological diagnosis of high-grade intraepithelial lesions and 10 healthy subjects admitted at the Imam Khomeini Hospital (Tehran) during the years 2012 to 2014. All participants completed a self-administered questionnaire including information about demographic features, cigarette smoking, alcohol consumption, sexual practice, and the numbers of sexual partners and signed a written informed consent. Oral cavity specimens, such as lip, tongue, floor of mouth, buccal, gingival, and hard palate were obtained from the patients after surgery, transported to the virology laboratory and stored in -70ºC. This study was approved by the Department of Virology, School of Medicine, Tarbiat Modares University, Tehran, Iran.

### DNA extraction

Extraction of total DNA was performed using a manual procedure. The manual method included organic extraction (phenol-chloroform) of the specimens. Briefly, cellular pellets were resuspended in 500 μl lysis solution containing 100 mM KCl, 10 mM Tris-HCl (pH 8.3), 2.5 mM MgCl_2_, 0.5% (v/v) Tween-20, and 0.5% (v/v) Nonidet P-40. Samples were incubated at 95ºC for 30 min, mixed for 2 min and digested with 50 μl proteinase K (20 μg/ul). After an overnight incubation at 56ºC, specimens were heated at 95ºC for 10 min to inactivate the proteinase K. The precipitated nucleic acid was pelleted by centrifugation at 10,000 ×g for 15 min, washed twice with 70% ethanol and air-dried. The specimens were resuspended in 30 μl sterile distilled water[14]. Then 100 ng of total extracted DNA was used for PCR amplifications and further analysis.

### Primer design

The E6 genomic sequences were obtained from the GenBank, aligned by MEGA version 4 and finally identified as relatively well-conserved regions from nucleotides 157 to 174 and 338 to 351 according to the HPV-16/-18 sequence. The appropriate E6 region for HPV-11/-6-related genotypes was located from nucleotides 234 to 257 and 365 to 385. Designing of primer was performed using the Oligo version 7 and Allele ID version 6 software.

### Construction of plasmids carrying E6 fragments

The 190-bp HPV-16/-18 E6 amplicons (157-351) were PCR amplified, using Taq polymerase and standard PCR conditions, from positive subjects; positive clinical specimen for HeLa cell genomic DNA as positive control for HPV-18. Both amplicons were cloned into pTZR57 vector (Fermentas, USA) according to the manufacturer’s instructions to obtain the relevant plasmids. The 150-bp HPV-6/-11 E6 amplicons were PCR amplified from positive clinical specimen, and cloning was carried out similar to HPV-16/-18 E6 amplicons. Purified plasmids were sequenced to verify the accuracy of the sequences and also were used to be tested for their sensitivity and specificity in the conventional PCR and derive standard curves related to the real-time PCR assay.

### Generation of sensitivity and specificity

To determine the sensitivities of the PCR assays, the prepared plasmids were quantified by UV spectroscopy, and the viral copy number was calculated. The 10fold serial dilutions of all HPV DNA-containing plasmids were made. For sensitivity, PCR was performed on a 10fold dilution of plasmids ranging from 10^1^ to 10^8^ copies/reaction. The specificity was tested by determining the ability of primers to discriminate plasmids with different HPV types. There was no specific signal for the independent real-time PCR assays (data not shown).

### Conventional PCR amplification

DNA extraction was assessed by PCR amplification of a 260-bp fragment of β-globin gene using the forward 5′-GAAGAGCCAAGGACAGGTAC-3′ and reverse 5′-CAACTTCATCCACGTTCACC-3′ primers, 50 μg DNA specimen, 1.5 mM MgCl_2_ (Merck Company, Germany), 0.2 mM each dNTP (Cinagen, Iran), 5 pmol each primer, and 1 U Taq polymerase (Cinagen, Iran). Amplification was carried out for 35 cycles (94°C for 30 s, 60°C for 45 s, 72°C for 45 s) after an initial denaturation step of 94°C for 5 min, on a Techne Thermal Cycler (Techno, Genius, England). The cycles were followed by a 5-min extension at 72°C, and the PCR product was visualized on a 1.5% agarose gel by ethidium bromide staining and UV photography (Uvidoc, BTS-20-M, EEC). Regions of HPV-16/-18 and HPV-6/-11 genomes were detected by PCR using the primers 5’-F: GATTTATTTGTGGTGT ATAGAGAC-3’ and 5’-R: TTCTGCTGGATTCAACG G-3’ and the primers 5’-F: ACACTTTGATTATGCTG GATA TGC-3’ and 5’-R: GCTTTATGAACCGTGCC TTGG-3’, respectively to amplify two segments of approximately 190 bp related to HPV-16/-18 and 150 bp related to HPV-6/-11. PCR mixture consisted of 10 pmol of each primer, 50 ng DNA specimen, 1.5 mM MgCl_2_ in final concentration, 0.2 mM each dNTP in final concentration, and 1 U Taq polymerase (Cinagen, Iran). Amplifications were done for 35 cycles the same as β-globin gene. Finally, to confirm the genotyping of E6-specefic PCR for each sample, the sequence analysis was obtained from BLAST (http://www.ncbi. nlm.nih.gov/blast).

### Sybr Green real-time PCR

Real-time PCR reactions targeting the E6 region of HPV-16, -18, -6, -11 were performed using SYBR Green Master Mix (TaKara, Japan). The master mix was prepared for all specimens, as well as for positive and negative control samples. Real-time master mix consisted of 1.5 mM MgCl_2_, 0.2 mM each dNTP, 1 Unit Taq polymerase, and SYBR Green dye (Takara, Japan). Thermal cycle conditions started with first denaturation at 95°C for 10 minutes, followed by 40 cycles of 95°C for 10 s, 60°C for 10 s, and 72°C for 30 s. For standard curves, real-time PCR was performed on a 10fold dilution series of the plasmids as described above.

### Statistical analyses

SPSS 18 for windows (SPSS Inc., Chicago, IL) was used for statistical analysis. Categorical variables, including HPV infection and OSCC grade were compared by the Chi-squared or fisher’s exact test. All *P* value <0.05 was considered statistically significant.

## RESULTS

### Confirmation of HPV-6, -11, -16, and -18 plasmids

**Fig.1:**
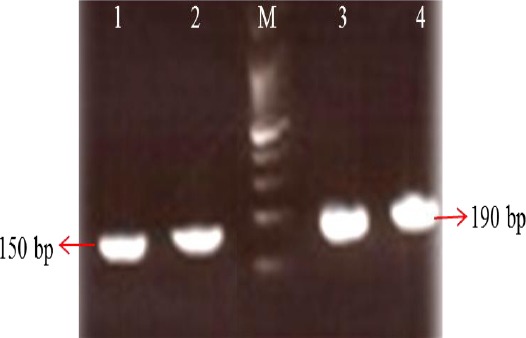
Amplification of the E6 gene by PCR method. Lanes 1 and 2, PCR products of HPV-6/-11 genotypes (150 bp); Lanes 3 and 4, PCR products of HPV-16/-18 genotypes (190 bp); M, 100 bp plus DNA size marker.

The HPV-6, -11, -16, and -18 E6 gene fragments were amplified by PCR, and the amplicons were ligated into pTZR57 cloning vector. The recombinant plasmids were then identified and confirmed by PCR using E6-specific primers (Fig. 1).

### Evaluation of sensitivity by conventional PCR

**Fig. 2:**
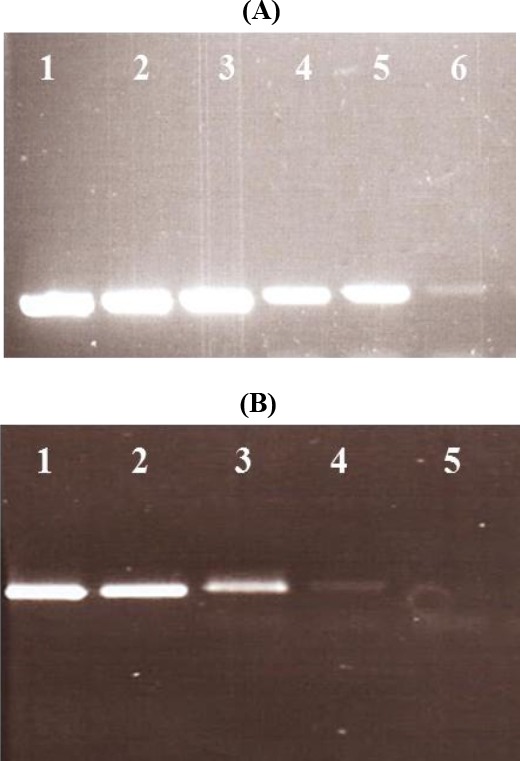
Determination of sensitivity of PCR method used in this study. Tenfold dilution series of HPV DNA-containing plasmids of types 6/11 and 16/18 were amplified. A significant sensitivity about the 100 viral copies was obtained for all four HPV genotypes. Depending on the genotypes 6/11 or 16/18, the amplification of the E6 gene produced a band of 150 bp or 190 bp, respectively. (A): Lanes 1-6, PCR products of 10^7^ to 10^2^ plasmid copy of HPV-6/-11. (B): Lanes 1-5: PCR products of 10^6^ to 10^2^ plasmid copy of HPV-16/-18.

The ability of all types of PCR primers to amplify HPV DNA-containing plasmids was analyzed by using a dilution series that started from 10^1^ plasmid copies and ended to 10^8^ plasmid copies. This assay showed significant sensitivity about 100 viral copies for all four HPV genotypes (Fig. 2).

### Viral load determination for HPV-11 and -18

In order to generate standard curves for analysis of HPV in samples, the DNA-containing plasmids were quantified by UV spectroscopy. Thereafter, a distinct set of 10fold dilutions for each construct was prepared and amplified by PCR in the same run of Real-Time PCR Machine (Rotor Gene 6000, Qiagene Inc., USA). A linear relationship between the virus copy number and Ct values was constructed for each of the HPV genotypes based on serial dilution associated to each plasmid. PCR assay on 50 clinical specimens showed that 18 (36%) were positive for HPV. Of these HPV-positive specimens, 10 (55.56%) and 8 (44.44%) were positive for HPV-18 and HPV-11, respectively. Table 1 shows DNA levels of HPV evaluated in 18 infected oral cavity specimens. As shown in the Table, 6 (33.32%) and 12 (66.65%) specimens were identified as high-titer and low-titer viral load, respectively.

**Table 1:** Human papillomavirus (HPV) DNA levels in 18 infected oral cavity specimens

HPV DNA (Copies/mL)	No. (% clinical specimens)		

	HPV-11	HPV-18	Total
Up to 1400	3 (16.66)	3 (16.66)	6 (33.32)
Up to 400	5 (27.77)	7 (38.88)	12 (66.65)
Total	8 (44.44)	10 (55.56)	18 (100)

## DISCUSSION

In 1982, Syrjanen reported his observation on correlation between HPV infectionand development of esophageal carcinoma[15]. This observation showed the presence of histological changes, which were consistent with those of condyloma in esophageal squamous cell cancer[16]. Several studies have also reported that HPV detection rate in esophageal squamous tumors is varied from 0% 100%[17,18,21]. This widespread variability may be explained by different factors such as disease status, sample collection methods, demographic and ethnic factors, and the sensitivity of the methods[17].

Regarding this fact that HPV is considered as an etiologic agent of oral squamous tumors, screening the patient with an increased risk of developing oral squamous tumors seems to be necessary[18]. There are major diagnostic methods in HPV detection and genotyping of clinical specimens. These techniques can be divided into three categories as follow: 1) target amplification, 2) signal amplification, and 3) probe amplification. PCR is one of the most examples of target amplification methods[1,19]. Previously, different HPV genotypes have been reported in Iran as well as other countries based on the DNA sequence of various regions of the viral genome, including capsid L1 gene[20].

Here, we have described for the first time in Iran a PCR-based method for the accurate and sensitive detection of high-risk genotypes (HPV-16/-18) and low-risk genotypes (HPV-6/-11) using the ur designed E6-specific primers. PCR-based detection of different types of HPV using the general or consensus primers necessitates confirming the underlying genotypes with time-consuming and costly techniques, such as sequencing, line immunoassay, restriction fragment length polymorphism, Southern-blot hybridization, or enzyme immunoassays. In addition, this method could potentially predict the progression of malignancy caused by high-risk HPV genotypes. In two different studies by Sotlar *et al*.[19] and Kenarkoohi *et al*.[20], PCR assay based on the detection of the viral E6/E7 oncogenes (as the primer-binding site) has been reported to be sensitive and specific for detection of 18 different HPV genotypes. Similar to Sotlar *et al*. [19], we found a high sensitivity range as much as 100 viral target copy for detection of high-risk (HPV-16/-18) and low-risk (HPV-6/-11) genotypes.

The result of the current investigation indicated that 36% (18 of 50) of clinical specimens tested were positive for HPV. Of these 18 investigated samples, 55.5% (10 of 18) were found to be positive for HPV-18, and the 45.5% (8 of 18) of the specimens were positive for HPV-11. In a recent study conducted by Tabatabai *et al*.[21], the frequency of HPV-related OSCC was 43%. In contrast to our study, they reported that HPV-16 is the most frequent type in patient group. Another study by Asvadi Kermani[25] detected 42.8% high-risk HPV types (16/18) in the studied patients, which is in line with our results that showed HPV-18 was the most frequent type in the case group.

According to the growing reports on the incidence of OSCC worldwide, the accurate determination of HPV levels in OSCC patients is serious. Real-time PCR has been shown to be a useful tool in quantifying HPV viral load[22,23]. In the present study, we analyzed the HPV DNA level in 18 (36%) HPV-infected oral cavity specimens by using the quantitative real-time PCR technique. We found that there is no significant correlation between the HPV status and OSCC tumor grade, which is consistent with the results reported by Saghravanian *et al*.[24]. In other study, Chen *et al*.[23] demonstrated that none of the patients with OSCC have been infected by HPV-16/-18. They also demonstrated that the real-time PCR following DNA sequencing is an effective approach to rule out false-positive specimens of HPV-16/-18. In general, these findings indicate that the gold standard method for identification of HPV is detection of viral DNA in clinical specimens.

PCR-based assay utilizing the E6 region, as primer-binding site, represents a highly sensitive and specific, rapid, and valid method for identification of HPV, especially the high-risk oncogenic HPV. The spectrum of this method could be extended by the addition of multiplex set of primers for other HPV genotypes. Furthermore, by using the real-time quantitative reverse transcription PCR for amplification of E6 oncogene transcripts, it may be possible to detect a further risk factor for the establishment or progression of a malignant lesion. This assay could also be employed as a potentially sensitive method in epidemiological and clinical follow-up studies, especially when exact HPV typing is required. Our results revealed that risk factors other than HPV infection play a possible role in OCSCCs among the Iranian population. Large-scale studies are needed to determine that HPV, as a risk factor, is independent of environmental factors such as alcohol and tobacco consumption, and sexual practices can be effective in creating OSCC.
